# Construction of white-rot fungal-bacterial consortia with improved ligninolytic properties and stable bacterial community structure

**DOI:** 10.1038/s43705-023-00270-4

**Published:** 2023-06-22

**Authors:** Toshio Mori, Taiki Terashima, Masaki Matsumura, Koudai Tsuruta, Hideo Dohra, Hirokazu Kawagishi, Hirofumi Hirai

**Affiliations:** 1 Faculty of Agriculture, Shizuoka University, 836 Ohya, Suruga-ku, Shizuoka, 422-8529 Japan; 2 Research Institute for Mushroom Science, Shizuoka University, 836 Ohya, Suruga‑ku, Shizuoka, 422‑8529 Japan; 3 Graduate School of Science and Technology, Shizuoka University, 836 Ohya, Suruga‑ku, Shizuoka, 422‑8529 Japan; 4 Research Institute of Green Science and Technology, Shizuoka University, 836 Ohya, Suruga-ku, Shizuoka, 422-8529 Japan

**Keywords:** Next-generation sequencing, Metagenomics, Fungal biology

## Abstract

It is believed that wood-rot fungi change their wood decay activities due to influences from co-existing bacterial communities; however, it is difficult to elucidate experimentally the interaction mechanisms in fungal-bacterial consortia because the bacterial community structure is quite unstable and readily changes. Indeed, the wood decay properties of fungal-bacterial consortia consisting of a white-rot fungus *Phanerochaete sordida* YK-624 and a natural bacterial community changed dramatically during several sub-cultivations on wood. Therefore, development of a sub-cultivation method that imparts stability to the bacterial community structure and fungal phenotype was attempted. The adopted method using agar medium enabled maintenance of fungal phenotypes relating to wood decay and the bacterial community even through dozens of repetitive sub-cultures. Some bacterial metabolic pathways identified based on gene predictions were screened as candidates involved in *P. sordida*–bacterial interactions. In particular, pathways related to prenyl naphthoquinone biosynthesis appeared to be involved in an interaction that promotes higher lignin degradation selectivity by the consortia, as naphthoquinone derivatives induced phenol-oxidizing activity. Based on these results, it is expected that detailed analyses of the relationship between the wood-degrading properties of white-rot fungal-bacterial consortia and bacterial community structures will be feasible using the sub-cultivation method developed in this study.

## Introduction

White-rot fungi are capable of degrading all major wood components, including cellulose, hemicellulose, and lignin. These organisms play an important role as saprotrophic decomposers in forest ecosystems. Fungi secrete a large array of enzymes to degrade both carbohydrates and lignin [1–3]. The ligninolytic reaction in particular is the most characteristic activity of white-rot fungi, as they are unique in their ability to mineralize lignin. White-rot fungi can be divided into two groups based on preference of lignin degradation in wood decomposition as selective lignin-degraders and non-selective degraders. Non-selective degraders decompose all major plant components simultaneously, whereas selective lignin-degraders prefer to degrade lignin over polysaccharides. Special ligninolytic enzymes, including lignin peroxidases, manganese peroxidases (MnPs), versatile peroxidases, and phenol oxidases, as well as the accessory enzymes involved in lignin degradation were reviewed by Dashtban et al. [1].

It is believed that white-rot fungi degrade dead wood in natural ecosystems through interactions with other microorganisms such as bacteria. Bacteria that interact with white-rot fungi can be classified based on their contribution to fungal-mediated wood decay. Some bacteria inhibit fungal wood decomposition, whereas other interacting bacteria stimulate fungal decomposition either directly or indirectly [4]. For example, microorganisms such as nitrogen-fixing bacteria and yeast are thought to stimulate fungal wood decomposition by enhancing fungal growth [5]. Other studies have reported that interactions between white-rot fungi and bacteria promote increased fungal growth [4]. There is little concrete information, however, regarding the mechanisms that promote lignin degradation via interactions between white-rot fungi and bacteria.

Some studies examining the composition of microbial communities in decaying wood have described bacterial taxa associated with wood-rot fungi (e.g., [6]). Bacterial diversity in decaying wood is heavily influenced by underlying soil and the effects of selection pressure from wood-inhabiting fungi, as the saprotrophic bacterial community is much less diverse than the soil bacterial community [6, 7]. Wood properties (e.g., pH, C/N ratio, water content, species) and stage of decay affect the composition of bacterial communities in decaying wood [4, 7]. Furthermore, Hervé et al. found that the composition of the bacterial community interacting with the white-rot fungus *Phanerochaete chrysosporium* exhibited stochastic differences between replicates [8]. Collectively, these data suggest that bacterial communities in decaying wood are relatively unstable and that the structure of the community changes during wood decay and replication processes.

The instability of bacterial communities is therefore one of the major bottlenecks in studies of the interactions between white-rot fungi and bacteria during wood decay. To elucidate the mechanisms of bacterial-fungal interactions, cultivation methods that provide good reproducibility of biological activity in bacterial-fungal communities are needed. In addition, bacteria that are unrelated to the bacterial-fungal interaction must be reduced or excluded from the microbial community in order to ensure that bacteria associated with white-rot fungi are dominant and that the microbial network complexity is minimized. Highly complex bacterial communities not only increase the difficulty of data analysis, they can also mask important target bacterial-fungal interactions due to bacterial-bacterial interactions. Ensuring that bacterial groups strongly associated with white-rot fungi, such as the genus *Burkholderia* [8], become stable and dominant should simplify the observation and elucidation of bacterial-fungal interactions. In the present study, therefore, we developed a cultivation method for consortia consisting of the white-rot fungus *Phanerochaete sordida* YK-624 and bacterial communities. This method promotes the stability of the bacterial community and fungal phenotype even with repetitive sub-culturing and enables estimation of interaction mechanisms that affect ligninolytic properties.

## Materials and methods

### Generation of fungal-bacterial consortia

The white-rot fungus *P. sordida* YK-624 (ATCC 90872) was pre-grown on potato dextrose agar (PDA). Mycelia appearing on PDA were then inoculated onto beech wood meal to create a fungal bed (6 × 6 × 5 cm), and the block was incubated for 1 month at 30 °C. After the mycelia spread, the block was placed into a plastic nonwoven bag and buried in soil in several locations around Shizuoka University, Shizuoka, Japan. The number of locations was four: two locations with forested soil (Ta and Tb), one location under humus (H), and one location with farm soil (M). The samples were left buried for 1–5 weeks (each sample was named with regard to the burial location and duration of burial as Ta3w, M1w, etc.). At the designated times, the blocks were recovered from soil, and the surface of each block was removed using a knife to obtain core samples. Each core sample was crushed uniformly and mixed with fresh beech wood meal (60–80 mesh) at a dry-weight ratio of 10% (moisture content adjusted to 70%). Plural mixed meals were prepared for each core sample and incubated for 2 weeks, after which the enrichment culture was mixed uniformly and thoroughly. A portion of the first sub-culture was dried at 105 °C for 1 day, and then the total weight loss (WL) was determined. The lignin content of the residue was measured by the Klason method, as described previously [9]. Lignin degradation (LD) was expressed as loss of total lignin from the calculated amount of lignin in the starting material. The remaining first sub-cultures were stored at 4 °C until use.

### Sub-cultivation on wood

Selected enrichment cultures (Ta1w, M2w, and M4w) were newly sub-cultured on beech wood meal for 4 weeks. The sub-cultures were then manually homogenized, and a portion (50 mg dry basis) was inoculated onto fresh beech wood meal (1 g, 60–80 mesh, moisture 70%). Sub-cultivation on wood was done repeatedly. These samples were designated with the combined sample name plus the time of sub-culture (e.g., M4w3, M4w5, etc.). Each sub-culture was evaluated for its wood-decay properties in the same manner described above.

### Attachment assay and sub-cultivation on PDAg

To separate bacteria attached to fungal hyphae and other bacteria, attachment assays were performed according to a method described in a previous report, with some modifications [10]. A small Petri dish was set into a large Petri dish to form a physical barrier. PDA containing 1.8 µM guaiacol (PDAg) was poured into both dishes, up to 5 mm below the edge of the smaller dish. A small portion of M4w3 wood culture was inoculated onto the center of the inner small dish. The dish was incubated at 30 °C until the hyphae spread to the medium in the outer dish and pigmented. The pigmented agar with mycelia was recovered from the inner and outer dish and sub-cultured repeatedly on PDAg. After pigmentation due to oxidation of guaiacol (7–8 days), a piece of mycelia on the pigmented PDAg (5 mm square) was cut and transferred to fresh PDAg. This procedure was repeated to create a series of sub-cultures on PDAg. These sub-cultures from the inner and outer dishes were designated M4w3pX and M4w3AApX (X indicates the time of sub-cultivation), respectively. These cultures were stored at 4 °C until use, then sub-cultured on PDAg at 30 °C to use further experiments. A mycelial disc (diameter 1 cm) obtained from PDAg cultures of M4w3p1, 10, 20, 30 and M4w3AAp1, 10, 20, 30 was inoculated onto beech wood meal (0.5 g, 80–100 mesh, moisture 70%) in a 50-ml Erlenmeyer flask and incubated at 30 °C. The disc was removed from the wood meal after 1 week and incubated for an additional 3 weeks. Subsequently, WL and LD rates were determined as described above. *Phanerochaete sordida* in single culture was used as a control according to the same experimental procedures. The selection factor (SF), an indicator of ligninolytic selectivity, was calculated as follows: SF = LD (%)/(WL [%] excluding lignin loss).

Crude enzyme solution was obtained as an effusion by freeze-thaw of the sub-cultures on PDAg. The effusion was filtered (0.2 µm pore size), and then low-molecular-weight compounds were removed using a PD-10 desalting column (GE Healthcare, Amersham Place, UK); 10 mM succinate-Na [pH 4.5] was used for equilibration and elution). The eluate was used to assay ligninolytic enzyme activity. MnP, lignin peroxidase, and phenol oxidase activity was measured as described previously [9]. Details of microscopic observation are shown in Supplementary information.

### Chemical induction of phenol-oxidizing activity

Dimethyl sulfoxide solutions of 1,4-napthoquinone (NQ) derivatives, 2-amino-3-chloro-NQ, 2-hydroxy-NQ (ACNQ and HNQ, Tokyo Chemical Industry Co., Ltd., Tokyo, Japan), and 2-methyl-NQ (MNQ, FUJIFILM Wako Pure Chemical Co., Osaka, Japan) at various concentrations were added to PDAg at 0.1% (v/v). The concentrations were adjusted to 4, 6, 8, 10, and 15 ppm with ACNQ; 50, 100, 250, and 500 ppm with HNQ; and 20, 40, 60, 80, and 100 ppm with MNQ. A PDAg pellet covered with *P. sordida* YK-624 mycelia was placed on each NQ-containing PDAg and incubated at 30 °C. Hyphal growth and pigmentation due to guaiacol oxidation were observed each day. A dimethyl sulfoxide -supplemented culture without NQs was used as a control.

### Diversity analysis

DNA was extracted from sub-cultures on beech wood (wet 100 mg) using Isoplant II (Nippon Gene Co., Ltd., Tokyo, Japan) following the product protocol (number of replicates were 2 for M4w1 or 3 for M4w3/5). An equal volume of 2× CTAB solution (1% cetyl trimethyl ammonium bromide, 2 M NaCl, and 1% polyvinylpyrrolidone) was added to extracted crude DNA solution (100 µl water) and incubated at 60 °C for 30 min. The mixture was then purified by chloroform extraction and isopropanol precipitation. This purification step was repeated twice. The mycelial pellets obtained from the sub-cultures on PDAg were inoculated onto a cellophane film (8.5 cm diameter) and placed on PDAg. After pigmentation following 7–8 days of incubation, mycelia spread on the film were recovered. DNA extraction buffer (450 µl) containing 100 mM Na-EDTA, 1.5 M NaCl, and 0.5 mg/ml lysozyme in 100 mM Tris-HCl (pH 8.0) was added to the mycelia and incubated for 30 min at 30 °C. Next, 100 µl of 10% SDS solution containing 0.5 mg/ml proteinase K was added to the solution and heated at 65 °C for 2 h with occasional gentle inversion. The resulting supernatant was purified with 2× CTAB solution as described above. DNA from farm soil was extracted following a protocol based on the direct lysis method of Zhou et al. [11].

Extracted DNA was finally purified using a DNeasy PowerSoil Pro kit (Qiagen, Hilden, Germany). The V3-V4 region of the 16S rRNA gene was amplified using the primers 341 F (5′-ACACTCTTTCCCTAC ACGACGCTCTTCCGATCT-NNNNN-CCTACGGGNGGCWGCAG-3′) and 805 R (5′-GTGAC TGGAGTTCAGACGTGTGCTCTTCCGATCT-NNNNN-GACTACHVGGGTATCTAATCC-3′) along with *ExTaq* DNA polymerase hot start version (TaKaRa Bio, Inc., Shiga, Japan). Library construction and sequencing were performed at Bioengineering Lab Co., Ltd. (Kanagawa, Japan).

The resulting amplicon sequences were processed by trimming low-quality ends using Trimomatic [12], removing primer sequences using TagCleaner [13], merging paired-end reads using Flash2 [14], and extracting 350–500 bp sequences using Seqkit [15]. Processed amplicon sequences were imported to QIIME2 (version 2022.2) [16] using the QIIME2 import plugin. The sequences were then demultiplexed and quality filtered using the q2-demux plugin, followed by denoising using the q2-dada2 plugin equipped with DADA2 [17]. Taxonomic assignment of the sequences was performed using the q2-feature-classifier plugin with a classifier pre-trained on the Ezbio database [18]. All sequences representing unassigned, archaea, eukaryote, and chloroplasts were filtered out using the q2-feature-table plugin. The final dataset for the downstream analyses included a total of 32 samples with 1808 features and a total frequency of 301,261 with minimum and maximum frequencies of 2084 and 37,869, respectively, whereas the mean frequency was 9414.4. Diversity analysis was performed using the q2-phylogeny and diversity plugins with a sample-depth of 2000 to measure the alpha diversity (Shannon and observed feature indexes). Permutation ANOVA (perMANOVA) analysis of the Bray–Curtis dissimilarities was carried out using 4999 unrestricted permutations for the bacterial community structure in the samples. Two-dimensional non-metric multidimensional scaling (NMDS) on Bray–Curtis distance matrix ordination plots of the bacterial community structure (abundance ratio base) across the different samples and redundancy analysis (RDA) were conducted using the ‘vegan’ R-package (vegan: Community Ecology Package. version 2.6-2) [19]. Distance matrixes were computed using an average of the bacterial community structure for each duplicated sample (consisting of the average of each bacterial taxon) using the ‘vegan’ R-package, and hierarchical clustering was then conducted. Prediction of metagenomic functional abundances based only on marker gene sequences was conducted using Phylogenetic Investigation of Communities by Reconstruction of Unobserved States (PICRUSt2) with a stratified option [20], and the summed per-genome pathway abundance was then obtained. Correlations between the difference in lignin degradation rates of consortia from the rate for single culture and pathway abundances predicted using PICRUSt2 were analyzed with the ‘multtest’ R-package (version 2.52.0) [21] to identify the bacterial pathway involved in lignin degradation. All raw reads of 16S rRNA gene amplicon sequences were deposited in the DDBJ Sequence Read Archive under the accession numbers SAMD00575319- SAMD00575350.

## Results

**Fig. 1:**
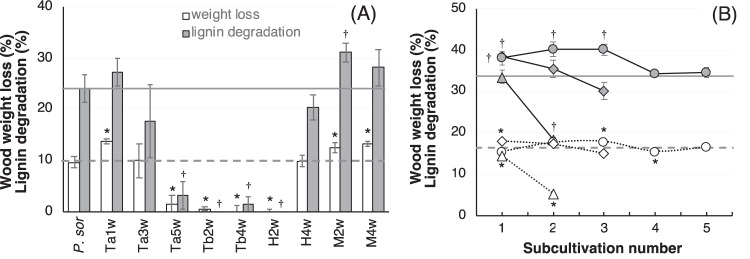
Wood-degradation properties of enrichment cultures and sub-cultures of *P. sordida–*bacterial consortia. **A** Wood weight loss (WL, white) and lignin degradation (LD, gray) in enrichment cultures after 2 weeks of incubation. **B** Effects of sub-cultivation with wood medium on WL and LD of the consortia. WL (white symbols) and LD (gray symbols) of M2w (triangles), M4w (circles), and Ta1w (rhombi) sub-cultures. WL and LD of *P. sordida* single cultures (*P. sor*) are indicated as dashed and solid lines, respectively. Asterisks (*) and daggers (†) indicate significant differences in WL and LD of consortia compared with those of *P. sordida* single culture, respectively (*p* < 0.05). Values are mean ± standard deviation of triplicate cultures.

Core samples obtained from buried fungal beds of *P. sordida* were cultured on wood meal to enrich for bacteria that coexist with the fungus. Enrichment cultures were then sub-cultured to evaluate the wood-degrading properties of fungal-bacterial consortia contained in the enrichment cultures. Rates of WL and LD after 2 weeks of incubation of enrichment cultures are shown in Fig. 1A. In comparison with *P. sordida* single culture, only three enrichment cultures, Ta1w, M2w, and M4w, showed higher WL rates. Some sub-cultures showed lower wood-degrading activity, suggesting that *P. sordida* was inhibited by exogenous microorganisms and lost its wood-degrading capability. Enrichment cultures that showed excellent wood-degrading properties were freshly sub-cultured on wood meal every 4 weeks. The rates of WL and LD of each sub-culture were measured to estimate the stability of the bacterial consortia (Fig. 1B). WL and LD decreased rapidly at every sub-culture stage in Ta1w and M2w. M4w sub-cultures retained high LD activity until the third sub-culture (M4w3); however, such a positive effect was lost after many repeated sub-cultivations. These results suggest that the microbial consortia in these cultures were unstable and that the diversity changed due to sub-cultivation on wood meal medium. Therefore, we attempted to develop a new sub-cultivation method that would exhibit excellent retention of the ligninolytic properties of fungal-bacterial consortia.

After several failures, we discovered that the M4w3 sub-cultures expressed phenol-oxidizing activity on PDAg, whereas pure *P. sordida* on the same medium did not show such a phenotype. On PDAg inoculated from the M4w3 sub-culture, hyphae covered 80% of the PDAg medium in a 9 cm petri dish within 2 days, and then pigmentation indicating guaiacol oxidation began. Pigmentation of the M4w3-inoculated culture expanded irregularly with increasing cultivation time, whereas no pigmentation was observed in *P. sordida* single culture over the same incubation period. This phenol-oxidation activity was retained regardless of the repetition of sub-cultivation or attachment assays (Fig. S1A). Crude enzyme solutions were extracted as effusions from PDAg on which the fungal-bacterial consortium M4w3p1 or *P. sordida* were grown, and the activity of ligninolytic enzymes in the extracts was measured. MnP activity was observed in the effusion from the consortia, whereas almost no activity was observed in the effusion from the *P. sordida* single culture (Fig. S1B). No lignin peroxidase or phenol oxidase activity was observed in extracts from the consortia or single cultures. In attachment assays, only bacteria attached to the fungal hyphae can overcome the physical barrier separating the fungal hyphae. Interestingly, pigmentation was observed in the outer medium of the attachment assay plates, suggesting that bacteria physically attached to the fungal hyphae induced *P. sordida* MnP enzyme activity. In addition, sub-cultures of the consortia obtained from the outer culture area of assay plates showed pigmentation due to guaiacol oxidation and MnP activity in the effusion (Fig. S1).

To confirm physical contact between bacterial cells and fungal hyphae, *P. sordida* single culture, M4w3p20, and M4w3AAp20 were observed using SEM. The *P. sordida* hyphae showed a smooth surface (Fig. S2A). However, a few spherical-shaped objects were observed on the fungal hyphae of M4w3p20 and AAp20 (Fig. S2B and C). Bacterial cells on M4w3AAp20 hyphae were then stained using a bacterial viability assay kit, which showed a rough hyphal surface and green fluorescence associated with live bacterial cells (Fig. S3). These morphologic characteristics and fluorescence were not observed in examination of hyphae from *P. sordida* single culture.

**Fig. 2:**
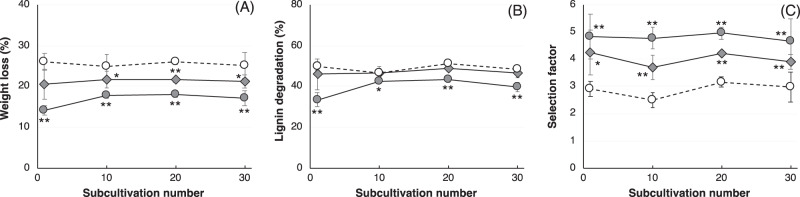
Wood-degrading properties of *P. sordida* single culture and M4w3p and M4w3AAp sub-cultures. **A** Wood weight loss, (**B**) lignin degradation, and (**C**) selection factor were measured using wood culture incubated for 4 weeks with *P. sordida* (open circles), M4w3p1-30 (closed rhombi), and M4w3AAp1-30 (closed circles) sub-cultures. Values are mean ± standard deviation of triplicate cultures. Asterisks (* and **) with the data points for M4w3p1-30 and M4w3AAp1-30 indicate significant differences from *P. sordida* single culture at the same number of sub-cultivations (*p* < 0.05 and <0.01, respectively).

The wood-degrading properties and bacterial diversity of each sub-culture of M4w3p1 to 30 (M4w3p sub-cultures) and M4w3AAp1 to 30 (M4w3AAp sub-cultures) were then characterized. In *P. sordida* single cultures, only minimal changes were observed in WL, LD, and SF, even after 30 sub-cultivations (Fig. 2A–C). Each M4w3p sub-culture exhibited almost identical wood-degrading properties, with slightly lower WL, the same level of LD, and clearly higher SF compared with *P. sordida* single culture. M4w3AAp sub-cultures also exhibited similar wood-degrading properties, with the exception of M4w3AAp1 sub-cultures, which showed lower WL and LD values. Much lower WL and LD values in addition to much higher SF values were observed in M4w3AAp10, 20, and 30 sub-cultures. After M4w3p19 was stored at 4 °C for several months, it was sub-cultured onto new PDAg to performed to a wood decay test. The wood decay properties were not shown significant difference in storage period within 12 month (Fig. S4). From this result, it seems that this cultures of microbial consortia can be stored for a long time and transported in a refrigerated state if care is taken to avoid drying and condensation. However, it will be necessary to consider detail of a preservation method to maintain the bacterial communities and effects of further subcultures in the future.

**Fig. 3:**
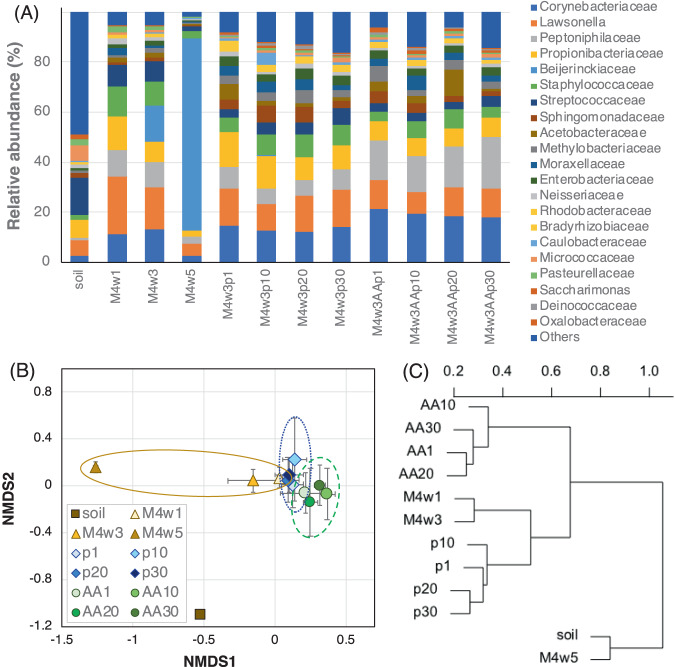
Comparative analysis of the bacterial community structure of each sub-culture. **A** Relative average abundances of phylogenetic groups (bacterial families) in each sub-culture (*n* = 2 or 3). “Soil” indicates farm soil at the fungal bed burial location (*n* = 1). “Others” indicates ASVs that could not be classified at the family level and ASVs with a relative abundance of 0.5% or less. **B** Two-dimensional non-metric multidimensional scaling (NMDS) ordination plots of bacterial community structures. Plots show centroids within each sub-culture, and bars represent one standard deviation along both NMDS axes. Brown, blue, and green circles indicate distribution ranges of M4w1-5, M4w3p1-30, and M4w3AAp1-30 sub-cultures, respectively. **C** 16S rRNA gene-based dendrogram generated using the Ward method based on distance matrixes of Bray–Curtis dissimilarities. The data designations “p1-30” and “AA1-30” indicate M4w3p1-30 and M4w3AAp1-30, respectively.

For phylogenetic analysis of bacteria in each fungal-bacterial consortium, bacterial DNA was extracted using the soft lysis method with detergent and enzymes. In amplicon sequences of the V3-V4 region of the 16S rRNA gene, none of the observed features reached the plateau of 2000 sequences (Fig. S5A); however, the Shannon index of the consortia seem to reach a plateau at a sequence depth of <500 (Fig. S5B). The Shannon index of the soil was slightly increased even beyond 2000 sequences. Thus, we concluded that diversity analysis of major amplicon sequence variants (ASVs) could be adequately carried out with 2000 sequences. Very low bacterial abundance relative to fungi or inadequate removal of PCR inhibitors may cause of the low numbers of sequences in some samples. As a result, it is possible that the major bacteria were preferentially detected. The relative abundances of the dominant bacterial families in soil, wood sub-cultures, and agar sub-cultures are shown in Fig. 3A. Bacterial diversity was clearly lower on wood sub-cultures from soil (Fig. S5). Corynebacteriaceae (mainly consisting of *Corynebacterium*), Lawsonella (*Lawsonella*), Peptoniphilaceae (*Anaerococcus* and *Finegoldia*), Propionibacteriaceae (*Cutibacterium*), Beijerinckiaceae (*Methylovirgula*), Staphylococcaceae (*Staphylococcus*), and Streptococcaceae (*Streptococcus*) were the major bacterial groups (relative abundance >5%) in M4w3. Although some bacterial groups showed differences in relative abundance between M4w3 and agar sub-cultures, most of the dominant taxonomic groups in M4w3 were also observed in M4w3p and M4w3AAp sub-cultures. The Beijerinckiaceae, including methanotrophs and aerobic nitrogen-fixing bacteria, were only observed in wood cultures; an excess abundance of this taxon in M4w5 was a possible cause of the reduced wood-degrading properties of these samples.

Changes in community structures resulting from sub-culturing on wood or agar were assessed using the primary and secondary axes of NMDS (Fig. 3B). Although marked changes in the community structures were observed during sub-cultivation on wood medium (between M4w3 and M4w5), the community structures in the agar sub-cultures (M4w3p1-30 sub-cultures and M4w3AAp1-30 sub-cultures) did not change significantly. A similar result was also shown in the perAMOVA analysis (Table 1) and its cluster dendrogram (Fig. 3C). No significant differences in community structure were observed among the samples within the M4w3p and M4w3AAp sub-cultures; however, significant differences were observed between the community structures of M4w3p and M4w3AAp sub-cultures. In contrast, the community structures in the M4w3p sub-cultures and M4w3 seemed to be relatively similar, but there were significant differences between the structures in M4w3AAp sub-cultures and M4w3. These results suggest succession of most of the microbial community occurred due to sub-culture from wood to agar medium and that the attachment assay can impact the community structure.

**Table 1:** Results of perMANOVA analysis of Bray–Curtis dissimilarities for bacterial ASV community structure between sub-cultures.

Group 1	Group 2	Sample size	pseudo-F^a^	*p* value^b^
M4w3p subcultures		11	0.476	0.98
M4w3p1	M4w3p10	5	0.474	0.895
	M4w3p20	5	0.453	0.907
	M4w3p30	5	0.528	0.794
M4w3p10	M4w3p20	6	0.483	0.896
	M4w3p30	6	0.521	0.902
M4w3p20	M4w3p30	6	0.392	0.705
M4w3AAp subcultures		12	0.518	0.993
M4w3AAp1	M4w3AAp10	6	0.671	0.798
	M4w3AAp20	6	0.312	1
	M4w3AAp30	6	0.527	1
M4w3AAp10	M4w3AAp20	6	0.519	0.711
	M4w3AAp30	6	0.59	0.913
M4w3AAp20	M4w3AAp30	6	0.512	1
M4w3 × M4w3p subcultures × M4w3AAp subcultures		26	2.069	0.006^*^
M4w3AAp subcultures	M4w3p subcultures	23	2.008	0.031^*^
	M4w3	15	2.484	0.005^*^
M4w3p subcultures	M4w3	14	1.726	0.069

^a^Pseudo-F = F value by permutation.

^b^*P* values are based on 4999 permutations. Asterisk indicates statistical significance (*P* < 0.05).

To elucidate the relationship between dominant ASVs (relative abundance >0.5%) and wood-degrading properties of the consortia, RDA was performed using averages of relative ASV abundances in agar sub-cultures (M4w3p1-30 and M4w3AAp1-30) and the rates of change in WL/LD in *P. sordida* single culture. However, no clear relationship was observed among all tested ASVs (Fig. S6). The whole community pathway (sum of stratified metabolic pathways) and functional abundances were then predicted based on the bacterial community structures of M4w3p and M4w3AAp sub-cultures using PICRUSt2 [20]. This was followed by analysis of the correlation between the predicted pathway abundances and the changes in ratios of wood-degrading properties. The pathways showed significant correlations in both WL and LD (*p* < 0.05), and Pearson product-moment correlation coefficients are shown in Table 2. The primary pathways showing correlations were general metabolic pathways, including nucleic acid–related compound metabolism, amino acids metabolism, and energy metabolism. We then focused on the biosynthetic pathways related to naphthoquinols that might play roles in wood degradation by the consortia (see Discussion). It should be noted that pathways PWY-5840, 5863, 5897, 5898, and 5899 include the entire PWY-5837 pathway, and the only difference between these pathways in the MetaCyc database (https://metacyc.org/) is in the synthetic pathways for prenyl side chains and conjugation of prenyl diphosphate and naphthoquinone. ASVs involved in these pathways were very similar; in particular, ASVs involved in PWY-5897 to -5899 were identical according to PICRUSt2 results.

**Table 2:** Predicted bacterial metabolic pathways correlated with changes in rates of wood weight loss (WL) and lignin degradation (LD) in the consortia.

BioCyc ID	WL		LD		Pathway
	rho	*p* value	rho	*p* value	
ARGORNPROST-PWY	0.934	0.001	0.852	0.007	L-arginine degradation (Stickland reaction)
GLUCONEO-PWY	0.897	0.003	0.84	0.009	gluconeogenesis I
P42-PWY	0.901	0.002	0.811	0.015	incomplete reductive TCA cycle
PWY-5022	0.756	0.03	0.78	0.022	4-aminobutanoate degradation V
PWY-5837	0.736	0.037	0.711	0.048	2-carboxy-1,4-naphthoquinol biosynthesis
PWY-5840	0.783	0.021	0.77	0.025	superpathway of menaquinol-7 biosynthesis
PWY-5863	0.782	0.022	0.756	0.03	superpathway of phylloquinol biosynthesis
PWY-5897	0.748	0.033	0.723	0.043	superpathway of menaquinol-11 biosynthesis
PWY-5898	0.748	0.033	0.723	0.043	superpathway of menaquinol-12 biosynthesis
PWY-5899	0.748	0.033	0.723	0.043	superpathway of menaquinol-13 biosynthesis
PWY-5910	0.723	0.043	0.725	0.042	superpathway of geranylgeranyldiphosphate biosynthesis I
PWY-7196	0.918	0.001	0.821	0.012	superpathway of pyrimidine ribonucleosides salvage
PWY-7199	0.86	0.006	0.756	0.03	pyrimidine deoxyribonucleosides salvage
PWY-7200	0.909	0.002	0.814	0.014	superpathway of pyrimidine deoxyribonucleoside salvage
PWY-922	0.722	0.043	0.724	0.042	mevalonate pathway I
RUMP-PWY	0.73	0.04	0.738	0.036	formaldehyde oxidation I
TEICHOICACID-PWY	0.779	0.023	0.77	0.025	poly(glycerol phosphate) wall teichoic acid biosynthesis
HSERMETANA-PWY	−0.722	0.043	−0.707	0.05	L-methionine biosynthesis III
POLYISOPRENSYN-PWY	−0.921	0.001	−0.908	0.002	polyisoprenoid biosynthesis (E. coli)
PWY-5100	−0.78	0.022	−0.714	0.047	pyruvate fermentation to acetate and lactate II
PWY-5659	−0.888	0.003	−0.868	0.005	GDP-mannose biosynthesis
PWY-5695	−0.825	0.012	−0.792	0.019	inosine 5′-phosphate degradation
PWY-5913	−0.902	0.002	−0.855	0.007	partial TCA cycle (obligate autotrophs)
PWY-6151	−0.737	0.037	−0.739	0.036	S-adenosyl-L-methionine cycle I
PWY-6317	−0.873	0.005	−0.851	0.007	D-galactose degradation I (Leloir pathway)
PWY-6397	−0.904	0.002	−0.856	0.007	mycolyl-arabinogalactan-peptidoglycan complex biosynthesis
PWY-6545	−0.816	0.013	−0.757	0.03	pyrimidine deoxyribonucleotides de novo biosynthesis III
PWY-6897	−0.889	0.003	−0.818	0.013	thiamine salvage II
PWY1G-0	−0.86	0.006	−0.81	0.015	mycothiol biosynthesis

Finally, the effects of NQ derivatives on fungal growth and phenol-oxidizing activity on PDAg were examined. NQ derivatives exhibited growth-inhibitory effects in the following order: ACNQ > MNQ > HNQ. The minimum inhibitory concentrations of ACNQ, MNQ, and HNQ were 10, 100, and 500 ppm, respectively (Table 3). Phenol-oxidizing activity was induced in *P. sordida* growing on PDAg by MNQ and HNQ at 40 and 50 ppm, respectively (Table 3). No pigmentation was observed in control (without NQs) and ACNQ-supplemented cultures. In summary, phenol-oxidizing activity was induced in *P. sordida* by NQs under low-toxicity conditions but suppressed under high-toxicity conditions.

**Table 3:** Effect of naphthoquinones (NQs) on the inhibition of growth and promotion of phenol-oxidizing activity of *P. sordida*.

Naphthoquinone	MIC (ppm)^a^	Pigmentation^b^	
		Concentration (ppm)	Incubation period (days)
ACNQ	10	–	–
HNQ	500	50	14
MNQ	100	40	17

^a^Minimum inhibitory concentration at which no mycelial growth was observed after 20 days of incubation.

^b^Concentration and incubation period at which pigmentation by phenol oxidation was observed in the PDAg containing each NQ.

## Discussion

The wood-degrading activity of wood-rot fungi is known to be affected by co-existing microorganisms. However, relatively little information exists regarding the mechanisms of interaction between wood-rotting fungi and bacteria that affect wood decay. This is probably because the diverse bacterial community is quite unstable, making experimental reproducibility difficult to achieve. Indeed, Hervé et al. reported that bacterial community structures change readily during replication on wood [8]. Their report also indicated that the bacteria community associated with wood was less diverse than the initial inoculum and changed with time and replication. In the present study as well, sub-cultivation on wood medium caused dramatic changes in the structure of the bacterial community in the consortium. It can be said that it is difficult to stably replicate the bacterial community structure of fungal-bacterial consortia on wood culture. Therefore, a new sub-cultivation method that maintains the bacterial community structure is needed to shed light on the relationship between the bacterial community and the phenotype of white-rot fungi. With an emphasis on the functional reproducibility, we examined several cultivation methods to maintain the wood-degrading properties and bacterial community structure of fungal-bacterial consortia, such as supplementation of the wood culture with nutrients and sub-culturing using liquid media. After much trial and error, we finally discovered that agar medium enables replication of the bacterial communities in consortia while maintaining properties such as phenol oxidation and high SFs. Agar medium enables better maintenance of wet conditions, nutrient contents, and pH during cultivation compared with wood culture methods. Because these characteristics support the growth of a wide range of microorganisms, agar sub-culture likely enables the passage of most major taxa of bacteria from wood sub-culture and maintenance of bacterial community structures even after repeated sub-cultivations. On the other hand, bacterial groups dependent on some factor on the decaying wood probably not be reflected in the community on agar culture, due to quite different conditions between wood and agar cultures. Although the bacterial community structures of the M4w3p and M4w3AAp sub-cultures appeared very similar at first glance, this sub-cultivation method maintained the statistical differences in the community structures through 30 sub-cultivations (Fig. 3B and Table 1).

M4w3p and M4w3AAp agar sub-cultures were obtained from the M4w3 wood sub-culture that showed higher ligninolytic activity than *P. sordida* YK-624 single culture. These agar sub-cultures maintained highly selective LD activity, although their WL was lower than that of the single culture (Fig. 2). Bacterial interactions with wood-rot fungi in decomposing wood were reviewed by Johnston and co-authors in 2016 [4]. According to their review, there are several reported cases of bacteria affecting the wood-degrading activity of wood-rot fungi, but the effects of bacteria are not consistent and vary depending on the environmental conditions, bacterial community structures, and species of wood-rot fungi. In several studies, bacterial communities have been analyzed in wood degraded by white-rot fungi, and their community structures were found to differ [8, 22**–**24]. Therefore, it is difficult to estimate relationships between the phenotype of white-rot fungi and bacterial community structures from current data. In addition, Johnston et al. reported that the promotion of wood decay can be attributed to an increase in fungal activity due to bacteria supplying nutrients, such as nitrogen, or to the responses of the wood-rotting fungi against other bacterial activities, such as antagonism [4]. Although nutrients supplied by bacteria are simple factors that promote wood decay and fungal growth, factors that support growth probably involve complex mechanisms consisting of microbial interactions between white-rot fungi and bacteria, but there is a considerable lack of information at this time.

Several reports have been published regarding bacterial communities in decaying wood and bacterial groups potentially related to white-rot fungi. A close association has been reported between Burkholderiaceae and fungi such as *P. chrysosporium* [8, 25], and fungal-specific Sphingomonadaceae have been found in mycospheres of basidiomycete fungi [26]. In the present study, Burkholderiaceae were not detected in soil where fungal beds were buried, and these organisms were rarely present in the consortia of our samples, whereas members of the Sphingomonadaceae (mainly consisting of *Sphingomonas*) were found in both the soil and consortia. Bacteria belonging to the Caulobacteraceae, Enterobacteriaceae, Methylobacteriaceae, and Sphingomonadaceae reportedly degrade lignin [27, 28]. Furthermore, nitrogen-fixing bacteria that harbor nitrogen dehydrogenase genes possibly present in decaying wood include Rhizobiales, Sphigomonadales, and Burkholderiales [4]. Rhizobiales (Beijerinckiaceae, Bradyrhizobiaceae, and Methylobacteriaceae), Sphingomonadales (Sphingomonadaceae), and Burkholderiales (Oxalobacteraceae) were also observed in the present study. These data suggest that Sphingomonadaceae bacteria in the consortia interact with *P. sordida*.

In investigations of the relationship between microbial community and the wood-degrading properties of the consortia using RDA biplots, no dominant ASVs related to these properties were found (Fig. S6). This result suggests that the properties of the consortia may be defined by the influence of minor and/or multiple bacterial strains. Therefore, PICRUSt2 was used to predict the metabolic pathway abundance of the bacterial communities in the consortia of our samples, and several metabolic pathways were correlated with the wood-degrading properties of the consortia (Table 2). Menaquinones and phylloquinones, which contain naphthoquinone in their structure, comprise one major group of bacterial isoprenoid quinones. Menaquinones are utilized in electron transport systems, are generally found in strictly anaerobic bacteria, *Mycobacteria*, and most gram-positive bacteria as the sole quinone, and phylloquinones are found in cyanobacteria instead of menaquinones and ubiquinones [29]. Facultative bacteria retain both menaquinones and ubiquinones for adaptation to anaerobic and aerobic conditions, respectively. Although these compounds are insoluble in water and normally found within a lipid bilayer, some bacteria release quinone-type compound(s) to the extracellular environment, such as *Bacillus subtilis* [30], *Lactococcus cremoris* mutants [31] and *Propionibacterium freudenreichii* [32]. In addition, quinones including naphthoquinones function in quinone redox cycling reactions in white-rot fungi and are involved in the production of extracellular reactive oxygen species such as O_2_^•-^, ^•^OH, and H_2_O_2_ that are candidates for wood degradation agents [33, 34]. Curreli et al. reported that plant-derived hydroxylated naphthoquinones, including HNQ (aka. Lawsone), inhibit mycelial growth and promote the activity of ligninolytic enzymes in the white-rot fungus *Pleurotus sajor-caju* [35]. These facts and our result (Table 3), suggesting that the mechanisms of growth inhibition and promotion of ligninolytic activity by bacterial and plant-derived NQs (or something other oxidation stress substances) are widespread among white-rot fungi.

For future study of interaction mechanisms between white-rot fungi and bacteria that affect lignin degradation, we generated consortia consisting of a white-rot fungus and a bacterial community that exhibits stable bacterial diversity and fungal phenotypes. Although consortia consisting of the white-rot fungus *P. sordida* and soil bacteria showed unstable wood-degrading properties and bacterial community structures during sub-cultivation on wood medium, consortia exhibiting stable bacterial community structures and higher LD selectivity were successfully obtained using agar medium for sub-culture. These consortia sub-cultured on agar medium primarily included bacteria belonging to the Caulobacteraceae, Enterobacteriaceae, Methylobacteriaceae, and Sphingomonadaceae, including possible groups of lignin-degrading bacteria. The results of metabolic system prediction analyses also suggested that naphthoquinones are involved in the induction of fungal ligninolytic enzymes, among others. Based on these results, we hope that detailed analyses of the relationship between wood-degrading properties of white-rot fungal-bacterial consortia and bacterial community structures will be possible. This relationship has not been examined in the past but will be possible by applying the replicative cultivation method used in the present study. However, it should be mentioned that the microbial consortia constructed by enrichment cultivation represents only a fraction of the natural bacterial community. Moreover, shotgun metagenomic analysis should be able to give more accurate prediction of the interaction mechanism than PICRUSt2. It is not known at this stage whether this method can be applied to other fungi or woody materials. We are currently conducting a study to identify factors involved in the induction of fungal lignin-degrading enzymes within the consortia. In future work, we will use the agar-sub-culture method to attempt to construct various stable white-rot fungal-bacterial consortia that harbor different bacterial communities and exhibit different wood-degrading properties. Data sets regarding fungal phenotypes, bacterial community structures, and metabolic systems will be generated to clarify the white-rot fungal-bacterial interaction mechanisms that affect lignin and polysaccharide degradation.

## Supplementary information

Supplementary Information

## Data Availability

The datasets generated during the current study are available in the DDBJ repository, https://ddbj.nig.ac.jp/resource/biosample/SAMD00575331-50.
