# Supplementary material for: Construction of white-rot fungal-bacterial consortia with improved ligninolytic properties and stable bacterial community structure
Source: ISME Commun. 2023 Jun 22;3:61. doi: 10.1038/s43705-023-00270-4 (PMC10287725; doi:10.1038/s43705-023-00270-4)
Supplement: Supplementary file 1 — Supplementary Information [file 43705_2023_270_MOESM1_ESM.pdf]

Supplementary Information

Title:

Construction of white-rot fungal–bacterial consortia with improved ligninolytic properties and stable bacterial community structure

Authors:

Toshio Mori, Taiki Terashima, Masaki Matsumura, Koudai Tsuruta, Hideo Dohra, Hirokazu Kawagishi, Hirofumi Hirai

Microscopic observation

A cover glass was placed on a part of the culture away from the center of the PDA plate, and *P. sordida* YK-624 or M4w3p1 was inoculated at the center position. After mycelia grew on the cover glass, they were stained using a LIVE/DEAD BacLight Bacterial Viability kit (Invitrogen, OR, USA). An Olympus BX51 fluorescence microscope (Tokyo, Japan) was used to obtain SYTO9 and propidium iodide images. For scanning electron microscopy (SEM), a cellulose acetate membrane was placed on the PDAg, and mycelia were inoculated. After mycelial growth, pieces of the mycelia that covered the membrane were cut and air-dried overnight under ambient atmosphere. The samples were sputter-coated with Au/Pd for 4 min before observation with a JCM-6000 plus NeoScope scanning electron microscope (JEOL Ltd., Tokyo, Japan).

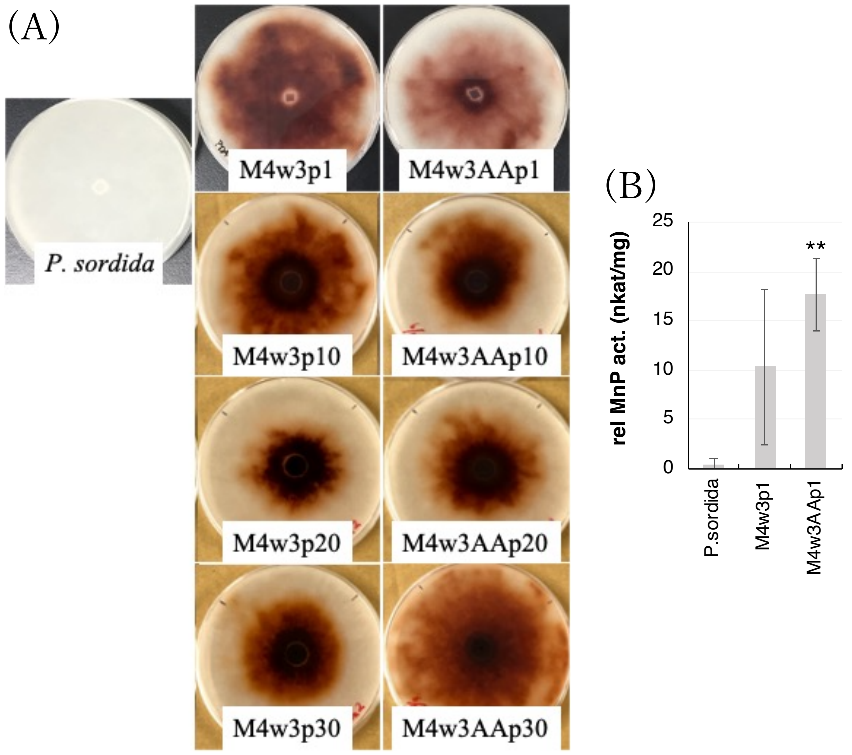

Figure S1 | Phenol-oxidizing activity of M4w3p and M4w3AAp sub-cultures on PDAg. (A) Pigmentation indicates guaiacol oxidation. (B) MnP activity in effluent obtained from PDAg cultures. Double asterisk (**) indicates a significant difference in MnP activity between *P. sordida* single culture and consortia ($p<0.01$). Values are mean ± standard deviation of triplicate cultures.

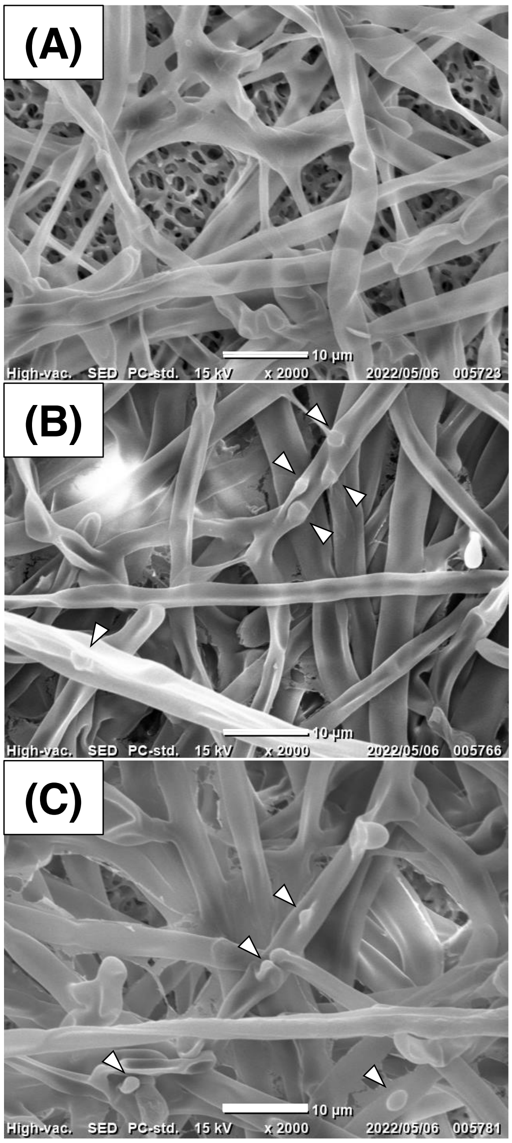

Figure S2 | Scanning electron microscopic photographs of hyphae from *P. sordida* and fungal-bacterial consortia. Panels show morphology of hyphae from *P. sordida* single culture (A) and M4w3p20 (B) and M4w3AAp20 (C) cultures. White arrowheads indicate spherical-shaped objects on hyphal surfaces. Scale bars are 10 µm.

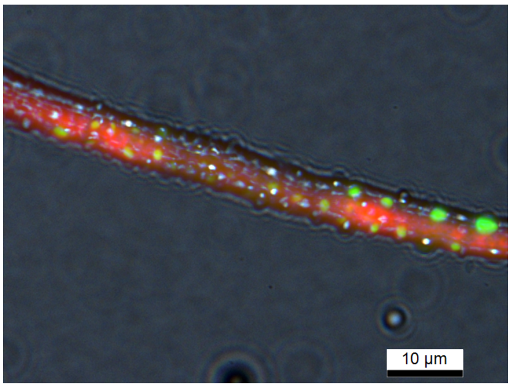

Figure S3 | Fluorescence microscopy photograph of a stained hypha grown in the M4w3p1 consortium. The hypha was stained using a LIVE/DEAD staining kit, and bright-field, SYTO9 (green), and propidium iodide (red) images were overlaid. Scale bar is 10 µm.

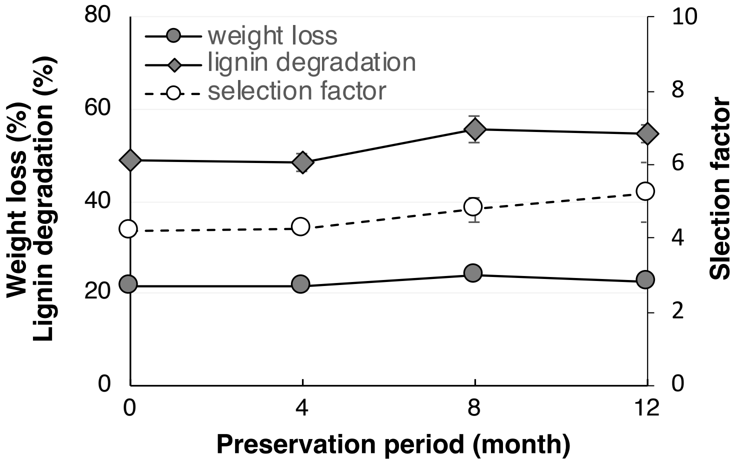

Figure S4 | Effect of storage period under refrigeration condition on wood decay properties of microbial consortia. Values are mean ± standard deviation of triplicate cultures. No significant difference was observed among respective analytical values of 1−12 month storage period by Tukey's test.

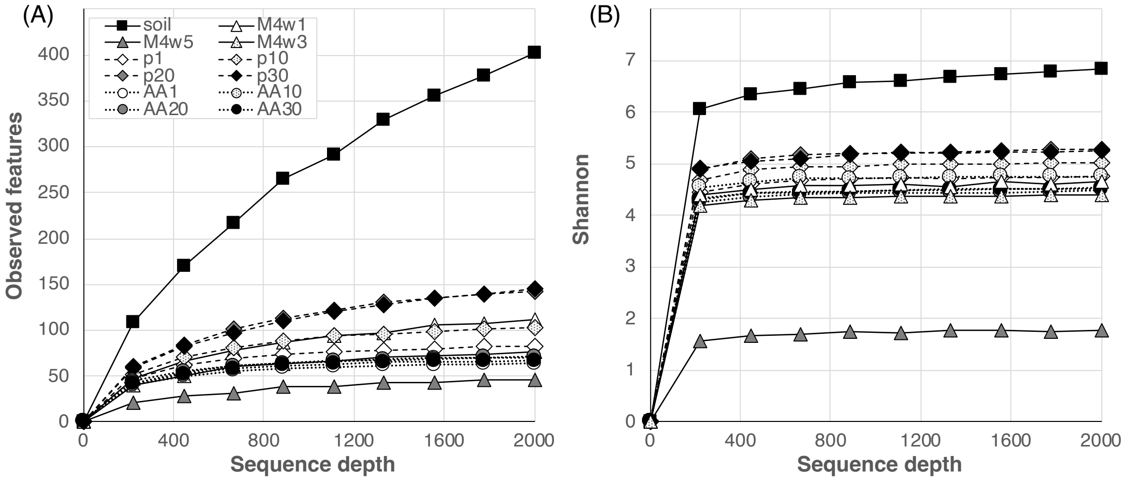

Figure S5 | Rarefaction curves for the number of observed features (A) and Shannon diversity indices (B). Soil, p1-30, and AA1-30 represent farm soil at the fungal bed burial locations, M4w3p1-30, and M4w3AAp1-30, respectively.

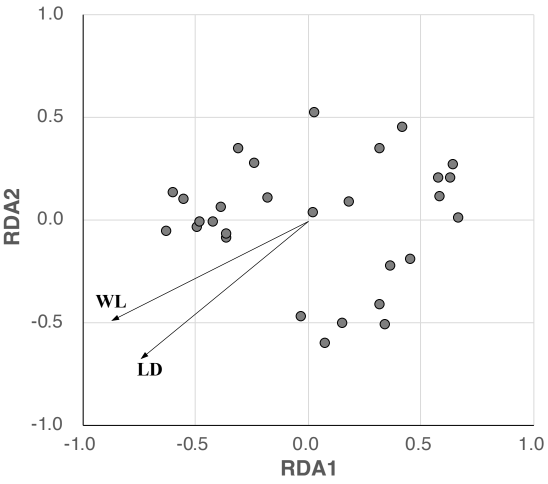

Figure S6 | Redundancy analysis (RDA) biplot representing the relationship between bacterial communities (ASVs) and wood-degradation properties determined in this study.
